# Spinal microglial β‐endorphin signaling mediates IL‐10 and exenatide‐induced inhibition of synaptic plasticity in neuropathic pain

**DOI:** 10.1111/cns.13694

**Published:** 2021-06-10

**Authors:** Le Ma, Shiyu Peng, Jinbao Wei, Mengjing Zhao, Khalil Ali Ahmad, Jinghong Chen, Yong‐Xiang Wang

**Affiliations:** ^1^ King's Lab Shanghai Jiao Tong University School of Pharmacy Shanghai China; ^2^ Shanghai Key Laboratory of Psychotic Disorders Shanghai Jiao Tong University School of Medicine, Shanghai Mental Health Center Shanghai China; ^3^ School of Life Sciences Westlake Institute for Advanced Study, Westlake University Hangzhou China

**Keywords:** β‐endorphin, exenatide, IL‐10, mEPSCs, microglia, mIPSCs, Neuropathic pain

## Abstract

**Aim:**

This study aimed to investigate the regulation of pain hypersensitivity induced by the spinal synaptic transmission mechanisms underlying interleukin (IL)‐10 and glucagon‐like peptide 1 receptor (GLP‐1R) agonist exenatide‐induced pain anti‐hypersensitivity in neuropathic rats through spinal nerve ligations.

**Methods:**

Neuropathic pain model was established by spinal nerve ligation of L5/L6 and verified by electrophysiological recording and immunofluorescence staining. Microglial expression of β‐endorphin through autocrine IL‐10‐ and exenatide‐induced inhibition of glutamatergic transmission were performed by behavioral tests coupled with whole‐cell recording of miniature excitatory postsynaptic currents (mEPSCs) and miniature inhibitory postsynaptic currents (mIPSCs) through application of endogenous and exogenous IL‐10 and β‐endorphin.

**Results:**

Intrathecal injections of IL‐10, exenatide, and the μ‐opioid receptor (MOR) agonists β‐endorphin and DAMGO inhibited thermal hyperalgesia and mechanical allodynia in neuropathic rats. Whole‐cell recordings of bath application of exenatide, IL‐10, and β‐endorphin showed similarly suppressed enhanced frequency and amplitude of the mEPSCs in the spinal dorsal horn neurons of laminae II, but did not reduce the frequency and amplitude of mIPSCs in neuropathic rats. The inhibitory effects of IL‐10 and exenatide on pain hypersensitive behaviors and spinal synaptic plasticity were totally blocked by pretreatment of IL‐10 antibody, β‐endorphin antiserum, and MOR antagonist CTAP. In addition, the microglial metabolic inhibitor minocycline blocked the inhibitory effects of IL‐10 and exenatide but not β‐endorphin on spinal synaptic plasticity.

**Conclusion:**

This suggests that spinal microglial expression of β‐endorphin mediates IL‐10‐ and exenatide‐induced inhibition of glutamatergic transmission and pain hypersensitivity via presynaptic and postsynaptic MORs in spinal dorsal horn.

## Graphical abstract

Schematic diagram shows the role of microglial expression of β‐endorphin in IL‐10‐ and specific GLP‐1 receptor agonist exenatide‐induced inhibition of spinal excitatory synaptic transmission and pain hypersensitivity in neuropathic pain. Following activation of GLP‐1 receptors, IL‐10 is released and then activates IL‐10 receptors via a microglial autocrine mechanism. Afterward, the β‐endorphin is released to microglial neuronal synapses and activates neuronal presynaptic and postsynaptic μ‐opioid receptors (MORs) to inhibit the enhanced glutamatergic transmission, leading to pain anti‐hypersensitivity.

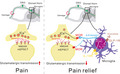

## 1 INTRODUCTION

Neuropathic pain is a major chronic pain disorder that arises from peripheral nerve injury typically characterized by abnormalities in sensation or reactions to stimuli, associated with neuronal loss, glial inflammation, and maladaptive nociceptive circuits.^1^ The rodent model of spinal nerve ligation (SNL) is popularly used to assay a variety of drugs for their therapeutic effects in neuropathic pain management and to determine the underlying cellular mechanisms. The unique axonal branches of the dorsal root ganglia could easily detect and immediately respond to injurious stimuli by transmitting information from the periphery to the brain through the spinal cord.^2^ Unlike neural circuits in the brain, spinal dorsal neurons, which are second‐order neurons in the process of nociception, receive dominated glutamate from primary afferent fibers and the descending inhibitory pathway from the higher brain regions, contributed to mediating and influencing nociceptive transmission.^3^ Nerve injury including rodent model of SNL may generate maladaptive dorsal horn plasticity and lead to pain states associated with alterations in N‐methyl‐D‐aspartate (NMDA) receptor‐mediated hypersensitivity, disinhibition of descending γ‐aminobutyric acid (GABAergic) /glycinergic inhibitory neurotransmission, and activation of glial cells, especially microglia.^2^, ^4^, ^5^, ^6^ The coordinative activity of microglia and neurons in brain diseases has been intensively studied,^7^, ^8^, ^9^ while microglial‐derived factors sensitize sensory processing through tumor necrosis factor (TNF)‐α, IL‐6, IL‐1β, and P2X inotropic receptors and leading to restore spinally mediated nocifensive reflexes mediated proinflammatory cytokines.^10^, ^11^, ^12^, ^13^ The importance of the interactions between microglia and the neural system has been increasingly recognized in the initiation and development of spinal plasticity in neuropathic pain, and targeting neuron‐microglia interactions have exerted the potential to effectively treat neuropathic pain.^7^, ^14^, ^15^, ^16^

Derived from microglia and other monocytes, IL‐10 is probably the most prominent anti‐inflammatory and immunosuppressive cytokine.^17^, ^18^ It also prevents damage to nerve injury and maintains neuronal homeostasis by modulating synaptic functions.^19^ For example, IL‐10 endowed anti‐inflammatory responses in STAT3 generated proinflammatory conditions, and dampen pathogenic Th17 cell responses and colitis.^20^ IL‐10 deficiency has preserved synaptic integrity and alleviated cognitive impairment in Alzheimer's disease models.^21^ Unconjugated bilirubin led to the overexpression of glutamate receptors and nitric oxide after neuronal damage accompanied by neurite outgrowth deficits. In primary neuronal cultures, IL‐10 exhibited profound neuroprotection correlated with modulation of neuronal morphogenesis and neuritic arborization.^22^ Our behavioral study indicated that stimulating exogenous and endogenous IL‐10 expression have displayed marked pain anti‐hypersensitive activity in animal models of neuropathic pain, peripheral diabetic pain, bone cancer pain, and complete Freund's adjuvant (CFA)‐induced inflammatory pain.^23^, ^24^, ^25^, ^26^, ^27^, ^28^ We also demonstrated that IL‐10’s anti‐neuropathic pain activity via the spinal microglia‐derived expression of β‐endorphin which depends on cAMP/PKA/p38β/CREB signaling separated from its spinal anti‐neuroinflammatory activity.^29^, ^30^, ^31^ In addition, recent study from our laboratory identified that activation of microglial expressed GLP‐1 receptors, α7 nicotinic acetylcholine (α7‐nACh) receptors, and G protein‐coupled receptor 40 (GPR40) effectively relieved pain states in a variety of rodent models of chronic pain through stimulating spinal microglial autocrine expression of IL‐10, IL‐10 receptors, and subsequent expression of β‐endorphin.^30^, ^32^, ^33^, ^34^, ^35^ Our data revealed that the microglial‐derived IL‐10/β‐endorphin represents a profound and broad pathway for pain transmission and transduction in regulation of pain hypersensitive states.

The imbalance of excitatory and inhibitory neurotransmission contributes to allodynia in neuropathic pain. Miniature excitatory or descending inhibitory postsynaptic currents may have great implications which reflect the synaptic functions of excitatory or inhibitory transmission coupled with retained synaptic structures and support the presynaptic and postsynaptic functions in spinal cord. The frequencies of mEPSCs are a notable feature of glutamate release from presynaptic membrane, while their amplitudes are associated with the density and functions of postsynaptic NMDA receptors.^36^, ^37^ Previous study of IL‐10 and GLP‐1 receptor agonists was typically focused on the antinociceptive effects and molecular signaling, while their functions in neuronal circuits of excitatory or descending inhibitory transmissions have not been illustrated in the spinal dorsal horn.

The aim of this study was to assess the effects and underlying mechanism of IL‐10 and the GLP‐1 receptor agonist exenatide on excitatory synaptic and descending inhibitory transmission in the spinal dorsal horn of neuropathic rats. We firstly characterized the L5/L6 spinal nerve ligated rat model of neuropathic pain by measuring long‐lastingly pain hypersensitive behaviors, and performed whole‐cell patch recordings of spinal excitatory synaptic and descending inhibitory neurotransmission in laminae Ⅱ neurons of spinal dorsal horn. We then tested the effects of IL‐10, exenatide, and exogenous β‐endorphin on pain hypersensitivity and excitatory and inhibitory synaptic transmissions of laminae Ⅱ neurons in neuropathic rats. Furthermore, the intervening agents such as agonists, antiserums, and antagonists were applied to determine whether β‐endorphin expression mediated exenatide‐ and IL‐10‐induced inhibition of spinal excitatory synaptic transmission. Lastly, using the microglial metabolic inhibitor minocycline, it exerted fully blockade effects of IL‐10‐ and exenatide‐induced inhibition of the frequency and amplitude of mEPSCs, without affecting β‐endorphin‐induced inhibitory effects. Our results indicate that IL‐10 and exenatide inhibit spinal synaptic plasticity through microglial expression of β‐endorphin, which contributes to their inhibition of hypersensitivity activity in neuropathic pain. Consequently, we further investigated the histological distribution of MOR in spinal dorsal horn through double immunofluorescent staining with presynaptic and postsynaptic markers. Together, these findings supported that IL‐10/β‐endorphin signaling regulates spinal excitatory synaptic transmission that abolish neuronal plasticity in neuropathic pain.

## 2 METHODS

### 2.1 Chemicals and reagents

Exenatide and β‐endorphin were synthesized from Dan Gang Peptides Co., the recombinant rat IL‐10, IL‐10 antibody and [D‐Ala2, N‐Me‐Phe4, Gly5‐ol]‐enkephalin (DAMGO) were obtained from Phoenix, R&D system, and Tocris respectively. Minocycline and D‐Phe‐Cys‐Tyr‐D‐Trp‐Arg‐Thr‐Pen‐Thr‐NH2 (CTAP) were purchased from Yuanye Biotech, picrotoxin (PTX), 6‐cyano‐7‐nitroquinoxaline‐2,3‐dione (CNQX) and D‐2‐amino‐5‐phosphonovaleric acid (D‐AP5) were obtained from Sigma‐Aldrich, tetrodotoxin (TTX) and strychnine were obtained from Aladdin and Toronto Research Chemicals, respectively.

### 2.2 Animals

To excluded the biorhythm cycle in female rats, male Wistar rats were used in this study.^38^ Rats were obtained from the Shanghai Experimental Animal Institute for Biological Sciences. Adult rats weighing 150–180 g were housed at a temperature of 23 ± 1℃ with a 12‐h light/dark cycle (lights on at 7:00 A.M.). Food and water were available *ad libitum*, and the rats were acclimatized to the laboratory environment for 7 days before the experiments were conducted. All experiments were performed in accordance with the Animal Care and Welfare Committee of Shanghai Jiao Tong University and followed the animal care guidelines of the National Institutes of Health (NIH Publications No. 8023).

### 2.3 Spinal nerve ligation and behavioral testing

Spinal nerve ligation (SNL) was performed as described previously.^39^, ^40^ Briefly, rats were anesthetized by intraperitoneal injection of pentobarbital sodium (50 mg/kg), and the left spinal nerves (L5/L6) were carefully displayed and ligated tightly with 6–0 silk sutures distally. Wounds were sutured strictly with 4–0 silk sutures. After 14 days of recovery, the rats with significant hypersensitivity to mechanical and thermal stimuli in operated side (mechanical paw withdrawal thresholds <8 g) and without any motor impairments were selected for further study. The rats were randomly assigned to each group. Compared with the SNL group, sham rats were operated on without L5 and L6 nerve ligation.

Before mechanical threshold testing, rats were well handled by investigators for at least 4 days,^41^ and were placed in a plastic box and acclimated individually for 30 min. A 2290 CE electrical von Frey hair (IITC Life Science), ranging from 0.1 to 90 g, was applied to measure the plantar surface of the rats’ hind paws until they suddenly withdrew, while the rats stood on the grid. The lowest force was recorded as the threshold. The procedure was repeated 3 times with a 1‐min interval. For thermal latency testing, rats were acclimated the atmosphere and transferred to an elevated plastic box on glass. Thermal threshold was determined using a 390G plantar test analgesic meter (IITC Life Science Inc.), the typical withdrawal behaviors were record as thermal threshold, and three withdrawal latency measurements were taken for each rat with a 5‐min interval. A 30‐sec cutoff was confined to prevent tissue damage, and the data were averaged for each test. All experiments were performed by investigators who were blinded to the treatments.

### 2.4 Intrathecal catheterization and injection in rats

A catheter with 0.28 nm inner diameter and 0.61 nm outer diameter (PE‐10, Clay Adams) was placed in the rat lumbar spine under inhaled isoflurane anesthesia as previously described.^42^ The rat lumber enlargements were determined by costal spinal angle and anatomical structure. Using man‐made joints, the catheter could tightly fix near the ilium and prevent from dislocated under the skin. The spinal nerve ligations were performed after intrathecal catheterization. The position of the catheter in the enlargement of spinal cord was verified when injection of 10 μl of 4% lidocaine in artificial cerebrospinal fluid (ACSF) followed by bilateral paralysis and claudication; the other rats were excluded from the study. All the rats were single housed to prevent the damage from other rats and without motor impairments were selected for further study after recovery for 14 days. For intrathecal administration, 10 μl of the drug solution was administered through a 50‐μl microinjector followed by a 15‐μl ACSF flush. All the catheters were confirmed after behavioral test in case of damages.

### 2.5 Immunofluorescence staining

The rats were anesthetized by intraperitoneal injection of pentobarbital sodium (50 mg/kg) and perfused with 50 ml ice‐cold 0.9% NaCl solution followed by 50 ml 4% paraformaldehyde (w/v) in pH 7.3 phosphate buffer saline (PBS), and spinal lumber enlargements (L3‐L5) were gently isolated and fixed overnight. The samples were then dehydrated with 30% sucrose in PBS (w/v) twice at 4℃, transferred into optimal cutting temperature compound (OCT) embedding agent (Leica Microsystems), and L5 segments were cut into 30‐μm slices. Spinal slices were stained by methods of bleach section. Spinal floating sections were incubated with 10% bovine serum albumin (BSA) (v/v) and 0.3% Triton X‐100 (v/v) for 1 h at room temperature (23 ± 1℃) with 40 revolutions per minute (rpm) ended by being washed three times for 5 min with 60 rpm in PBS. Spinal slices were incubated with various primary antibodies against c‐fos (1:1000, rabbit, SYSY) for immediate early genes, Bassoon (1:300, mouse, Abcam) for presynaptic marker, neuronal nuclear protein (NeuN) (1:300, mouse, Millipore) for neuron, glial fibrillary acidic protein (GFAP) (1:300, chicken, Millipore) for astrocyte, ionized calcium‐binding adaptor molecule‐1 (Iba‐1) (1:300, goat, Abcam) for microglia, postsynaptic density protein 95(PSD‐95) (1:100, mouse, Santa Cruz) for postsynaptic marker, and MORs (1:300, rabbit, Abcam) in PBST at 4℃ for 18 h with 40 rpm. The secondary antibodies (1:500, anti‐rabbit/mouse/chicken/goat Alexa‐488/568, Invitrogen) were incubated for 2 h at room temperature in 0.3% PBST with 40 rpm and washed 3 times with 5‐min intervals in PBS. All the slides were covered with dapi‐fluoromount‐G (Southern Biotech) and storage at 4℃ before acquisitions. For quantification of the intensity of biomarkers of neuron, astrocyte, and microglia in spinal dorsal horn, the parameters of each group were set to be identical. Immunofluorescence intensity measurements from positive staining areas of spinal dorsal horn were analyzed by FV10‐ASW 4.2 viewer. FV10‐ASW 4.2 viewer was also used to generate merged‐images in which colocalized areas appeared as yellow.

### 2.6 Spinal slice preparation

Spinal slices obtained from the rats 14 days after surgery were used for electrophysiological assessments. All the rats were tested by mechanical threshold testing and signed with numbers before recording. Researchers were blinded to the rats. Under inhaled isoflurane anesthesia, the L3‐L5 spinal cord was passively separated from the vertebrae and quickly immersed in ice‐cold oxygenated high‐sucrose ACSF containing (in mM): 234 sucrose, 3.6 mM KCl, 1.2 mM MgCl_2_, 1.2 mM NaH_2_PO_4_, 12 mM glucose, 2.5 mM CaCl_2_, and 25 mM NaHCO_3_ for 90 seconds. The L3‐L5 spinal cord was mildly blew with 50 mL syringe and immersed in ice‐cold ACSF. The spinal cords were covered by 2% agarose, and the 400‐μm spinal slices were cut on a vibratome (Leica VT‐1200S, Wetzlar, Germany), and more importantly, forceps were used to sign the other sides to distinguish the operated sides. The spinal slices were transferred and incubated in an oxygenated artificial cerebrospinal fluid (ACSF: 125 mM NaCl, 3 mM KCl, 1.25 mM NaH_2_PO_4_, 26 mM NaHCO_3_, 1 mM MgCl_2_, 2 mM CaCl_2_, and 10 mM D‐glucose, pH 7.3) for 30 minutes at 32℃ and cooling to room temperature for one hour, and then transferred to the recording chamber. Data acquisition was conducted by using an Axonpatch 200 B amplifier (Axon Instruments), and data were filtered at 2 kHz and digitized at 5 kHz using pClamp10 software.^43^

### 2.7 Whole‐cell recordings

The whole‐cell recording was conducted in the ipsilateral dorsal horn neurons of lamina II spinal slices with a pipette (4–5 MΩ). The pipettes were pulled from patch electrodes (1.0 mm outer diameter, 0.5 mm inner diameter; Sutter Instruments) with a horizontal puller (P‐97, Sutter Instruments) and filled with interval solution (140 mM Cs‐gluconate, 10 mM Hepes, 1.1 mM EGTA, 2 mM MgCl_2_, 3 mM MgATP and 0.3 mM Tris–guanosine triphosphate, pH 7.4 adjusted with CsOH), and mEPSCs were recorded after application of 0.5 µM TTX, 100 µM PTX, and 1 µM strychnine, and each rat was recorded for 3 cells at most for analysis. The neurons were voltage clamped at −70 mV, and the current traces were recorded for 5 minutes for analysis.

In addition, mIPSCs were recorded by high‐Cl^−^ interval solution (140 mM CsCl, 1.1 mM EGTA, 10 mM Hepes, 2 mM MgCl_2_, 3 mM MgATP and 0.3 mM Tris–guanosine triphosphate, pH7.4 adjusted with CsOH) clamped at −70 mV after bath application of TTX (0.5 µM), CNQX (20 µM), and D‐AP5 (50 µM) to block NMDA, kainate, and AMPA receptor activities.^44^ Access resistance of ≤30 MΩ was considered acceptable in whole‐cell recording. Clampfit 10.7 (Molecular Devices) used to calculate the cumulative distribution plots for mEPSCs and mIPSCs amplitudes and inter‐event intervals.

### 2.8 Data evaluation and statistics

GraphPad Prism 7.0 (GraphPad Software) was used for data analysis. The data were analyzed using two independent sample Student's *t* test, one‐way or repeated‐measures two‐way ANOVA, followed by Sidak's post‐tests for multiple comparisons. The results are represented as mean ± standard error of the mean (SEM). Before statistical analysis, the data were tested for Gaussian distribution assessed using Shapiro‐Wilk normality test after transform to logarithms. Furthermore, the data failed to show Gaussian distribution has been assessed by nonparametric tests. *p* values <0.05 were considered statistically significant. All the data were processed by CorelDraw 2019.

## 3 RESULTS

### 3.1 Phenotypic characterization of neuropathic pain

**FIGURE 1:**
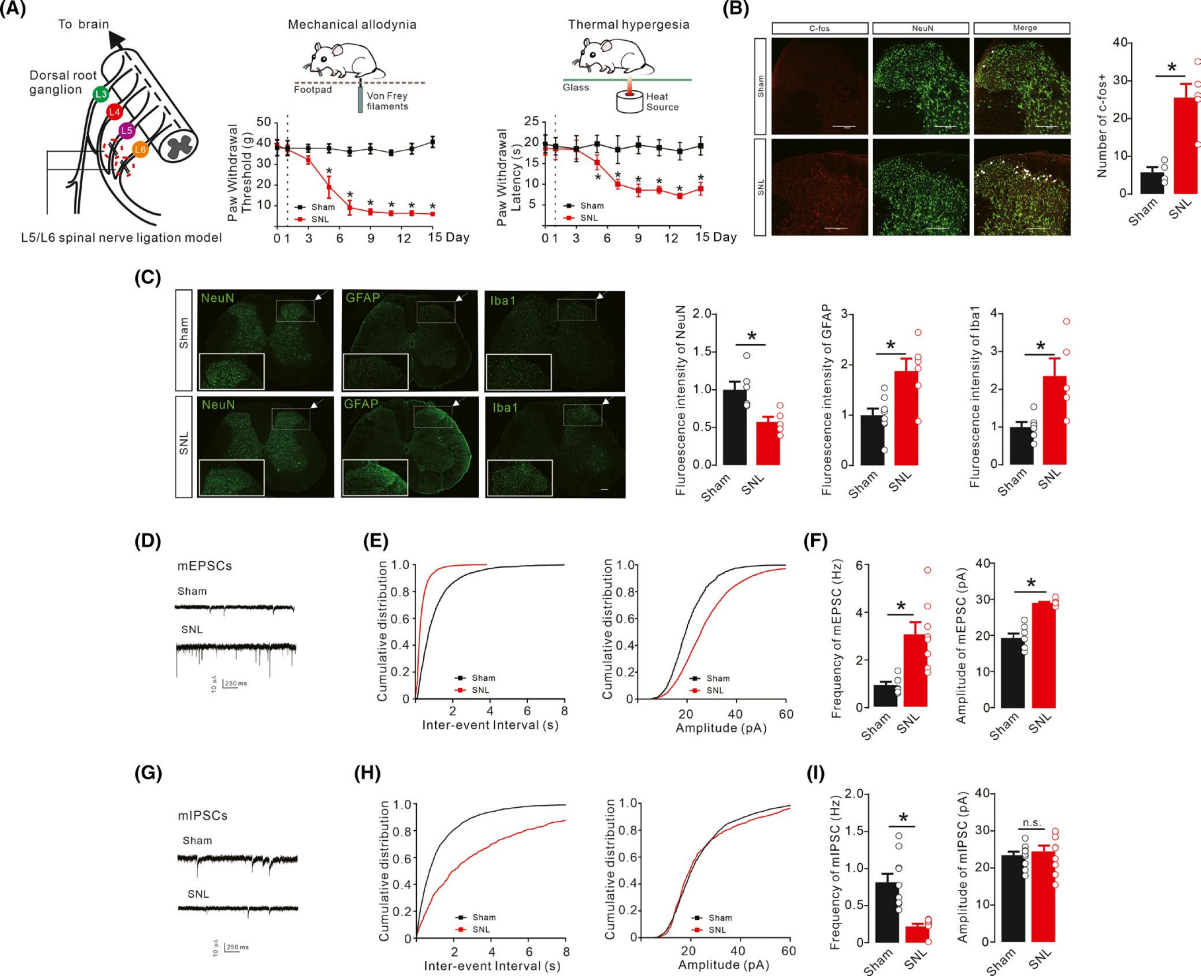
Mechanical allodynia and thermal hyperalgesia (A), increased neuronal c‐fos expression (B), regulation of astrocytic, microglial, and neuronal biomarkers GFAP, Iba‐1, and NeuN (C), enhanced excitatory synaptic transmission (D‐F), and reduced inhibitory transmission (G‐I) in the ipsilateral spinal dorsal horns of neuropathic rats. Neuropathic rats were induced by ligation of spinal nerves. Representative images and quantitative measurement of immunofluorescence staining of astrocytic, microglial, and neuronal biomarkers GFAP, Iba‐1, and NeuN and double immunofluorescence staining of c‐fos/NeuN in the ipsilateral spinal dorsal horns of lamina II of sham and neuropathic rats. Scale bar, 200 μm. Representative recording traces of mEPSCs and mIPSCs and cumulative distribution and statistical analyses were obtained from the lamina II spinal dorsal horn neurons. The data are presented as the means ± SEM (n = 4–8 animals). *p < 0.05, by unpaired and two‐tailed Student's *t* test or repeated‐measures two‐way ANOVA followed by Sidak's post‐tests

Peripheral nerve injury‐induced neuropathic pain in animals is characterized by alterations in synaptic transmission followed with abnormalities in pain sensation or response to stimulus.^4^, ^45^, ^46^, ^47^ This study firstly assessed the time courses of mechanical allodynia and thermal hyperalgesia in L5/L6 spinal nerve ligation induced neuropathic rats. Compared to sham rats, neuropathic rats exhibited time‐dependent thermal hyperalgesia and mechanical allodynia to innocuous mechanical and radiant stimuli in the ipsilateral hind paws, with peak effects 9 days after surgery that were maintained for 14 days (Figure 1A, mechanical allodynia *F*_(1,90)_ = 1635, *p* < 0.0001; thermal hyperalgesia *F*_(1,90)_ = 254.2, *p* < 0.0001, two‐way repeated‐measures ANOVA followed by Sidak's post‐tests). The SNL rats 14 days after surgery were then chosen for further tests.

In neuropathic rats, spinal nerve injury stimulated sensitization of neuronal circuits and upregulated the expression of c‐fos in the ipsilateral spinal dorsal horn neurons (laminae I/II) stimulated by innocuous mechanical stimuli after 1.5 h. Double immunofluorescence staining of c‐fos protein as a marker for neuronal activity with the neuronal biomarker NeuN was upregulated from 5.750 ± 1.377 in sham group to 25.60 ± 3.6 in neuropathic rats (Figure 1B). Consistent with previous studies,^29^, ^48^ SNL significantly upregulated the intensity of immunofluorescence staining of the biomarkers of microglia (Iba‐1) and astrocytes (GFAP) in the ipsilateral spinal dorsal horns compared to sham rats. However, neurons were declined (Figure 1C, in neuron, *t*_9_ = 3.211, *p* = 0.0106; in astrocyte, *t*_12_ = 3.387, *p* = 0.0054; in microglia, *t*_9_ = 3.028, *p* = 0.0143, two‐tailed unpaired *t* test).

To elucidate abnormalities in spinal excitatory synaptic transmission 14 days after nerve injury, patch‐clamp recordings of mEPSCs were conducted in the spinal ipsilateral slices from sham and neuropathic rats. The mEPSCs appeared as inward currents in the recorded neurons of the ipsilateral spinal dorsal horn (lamina II) (Figure 1D). Patch‐clamp recordings showed that both frequency and amplitude of mEPSCs were significantly increased compared with sham group (Figure 1E,F, Frequency, *t*_13_ = 3.860, *p* = 0.0020; Amplitude, *t*_13_ = 8.124, *p* < 0.0001, two‐tailed unpaired *t* test), which was in agreement with the previous finding of excessive glutamatergic synaptic transmission in pain hypersensitivity states.^49^, ^50^

Disinhibition is also known to contribute to pain hypersensitive states.^49^, ^51^ TTX, CNQX, and D‐AP5 were used to block glutamate transmission and sodium channels; patch‐clamp recordings of mIPSCs were acquired with high CI^−^ interval solution and held at −70 mV to assay the inhibitory neurotransmission. The results showed as inward currents in neurons of the ipsilateral spinal slices (lamina II) of sham and neuropathic rats (Figure 1G). The mIPSCs reflect descending GABAergic/glycinergic inhibitory transmission.^52^, ^53^, ^54^ Compared to the sham group, the frequencies but not magnitudes of mIPSCs in the ipsilateral neurons were significantly suppressed in the SNL group (Figure 1H,I, Frequency, *t*_18_ = 5.084, *p* < 0.0001; Amplitude, *t*_18_ = 0.5485, *p* = 0.5485, two‐tailed unpaired *t* test).

### 3.2 Reduced excitatory synaptic transmission mediated IL‐10‐induced pain anti‐hypersensitivity.

**FIGURE 2:**
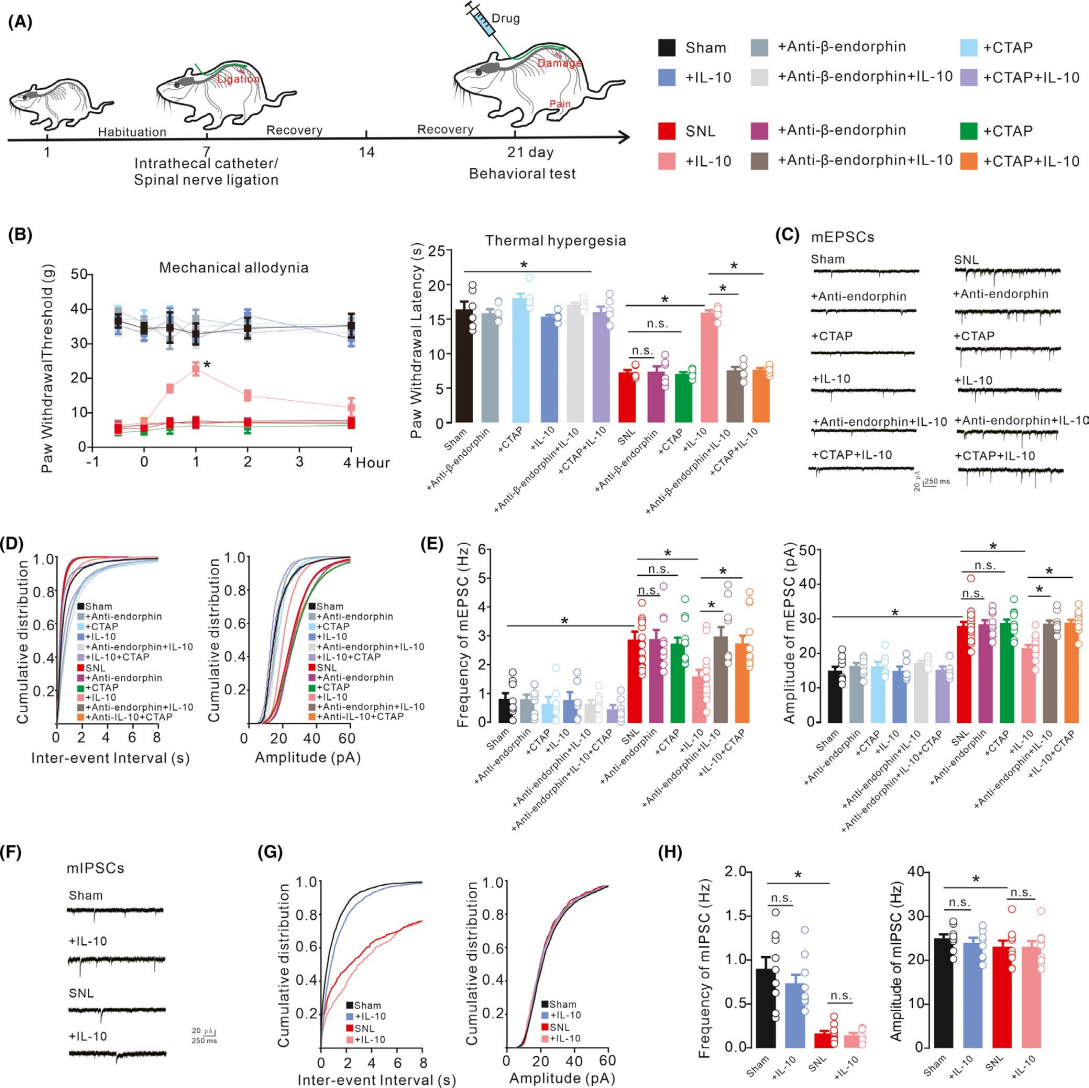
Schematic diagram showed the time procedures for spinal nerve surgery and drug administration (A). Blockade effects of the β‐endorphin antiserum and μ‐opioid receptor (MOR) antagonist CTAP on IL‐10 mechanical antiallodynia and thermal antihyperalgesia in the ipsilateral hind paws (B), inhibition of spinal excitatory synaptic transmission (C‐E), and effects on inhibitory synaptic transmission (F‐H) in neuropathic rats. Neuropathic rats were induced by ligation of spinal nerves. Representative mEPSCs and mIPSCs traces, cumulative distribution and statistical analyses were obtained from the lamina II spinal dorsal horn neurons. The data are presented as the means ±SEM (n = 5–6 animals). *p < 0.05, by one‐way or repeated‐measures two‐way ANOVA followed by Sidak's post‐tests)

In neuropathic rats, intrathecal delivery of IL‐10 has been shown to reduce pain hypersensitivity via spinal expression of β‐endorphin.^23^, ^30^, ^31^ To illustrate the role of spinal β‐endorphin expression in the reduction of excitatory synaptic transmission, the β‐endorphin antiserum and specific MOR antagonist CTAP were employed. The schematic diagram shows the experimental procedures performed on both sham and neuropathic rats for testing mechanical allodynia and thermal hyperalgesia following intrathecal injections of ACSF (10 μl), β‐endorphin antiserum (1:10), or CTAP (10 μg) followed by intrathecal delivery of ACSF (10 μl) or IL‐10 (100 ng) 30 min later (Figure 2A). Intrathecal delivery of 100 ng IL‐10 significantly inhibited thermal hyperalgesia and mechanical allodynia in the operated hind paws of rats, while the withdrawal responses in the hind paws of sham rats were not affected 1 h after the second intrathecal administration. Intrathecal pretreatment with β‐endorphin antiserum and CTAP did not significantly change baseline withdrawal responses in sham or neuropathic rats, but completely abolished IL‐10‐induced inhibition of mechanical antiallodynia and thermal antihyperalgesia in neuropathic rats (Figure 2B, mechanical allodynia, *F*_(11,360)_ = 1804, *p* < 0.0001, two‐way repeated‐measures ANOVA followed by Sidak's post‐tests; thermal hyperalgesia, *F*_(9,50)_ = 58.86, *p* < 0.0001, one‐way ANOVA followed by Sidak's post‐tests).

To further explore the mechanism of synaptic transmission in the spinal dorsal horn, excitatory synaptic transmission in the lamina II spinal dorsal horn neurons was recorded from both sham and neuropathic rats (Figure 2C). The frequency and amplitude of mEPSCs were significantly increased in the spinal dorsal horn neurons of neuropathic rats compared with sham rats. Bath application of IL‐10 (100 ng/ml) reduced the increased frequency and amplitude of mEPSCs from the SNL rats but not sham rats. Bath application of the β‐endorphin antiserum (1:300 dilution) or CTAP (1 μM) did not significantly change baseline mEPSCs in the sham group. However, it led to a complete recovery of the IL‐10‐inhibited frequencies and amplitudes of mEPSCs in the SNL group (Figure 2D,E, Frequency, *F*_(11,112)_ = 16.71, *p* < 0.0001; Amplitude, *F*_(11,112)_ = 35.40, *p* < 0.0001, one‐way ANOVA followed by Sidak's post‐tests).

To examine the effects of IL‐10 during pain states on inhibitory neurotransmission, whole‐cell recording of mIPSCs was applied (Figure 2F). However, bath application of IL‐10 (100 ng/ml) did not alter the frequency or amplitude of mIPSCs in either the SNL rats or sham rats (Figure 2G,H, Frequency, *F*_(3,32)_ = 19.52, *p* < 0.0001; Amplitude, *F*_(3,32)_ = 0.5984, *p* = 0.6207, one‐way ANOVA followed by Sidak's post‐tests).

### 3.3 Reduced excitatory synaptic transmission mediated exenatide‐induced pain anti‐hypersensitivity.

**FIGURE 3:**
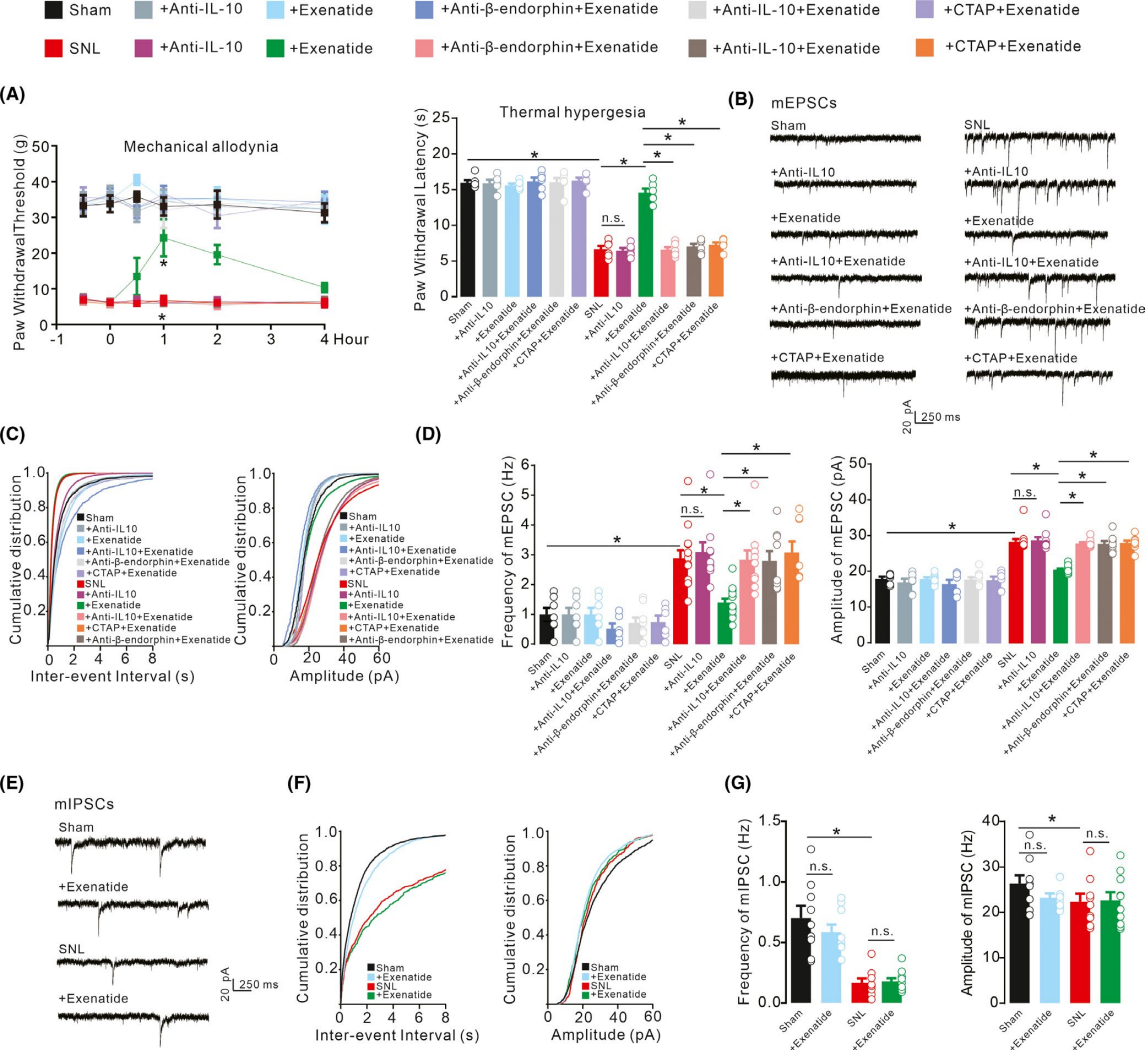
Blockade effects of the IL‐10 antibody, β‐endorphin antiserum, and μ‐opioid receptor (MOR) antagonist CTAP on the GLP‐1 receptor agonist exenatide‐induced inhibition of mechanical allodynia and thermal hyperalgesia in the ipsilateral hind paws (A), inhibition of spinal excitatory synaptic transmission (B‐D), and effects on inhibitory synaptic transmission (E‐G) in neuropathic rats. Neuropathic rats were induced by ligation of spinal nerves. Representative mEPSCs and mIPSCs traces, cumulative distribution and statistical analyses were obtained from the lamina II spinal dorsal horn neurons. The data are presented as the means ±SEM (n = 5–6 animals). *p < 0.05, by one‐way or repeated‐measures two‐way ANOVA followed by Sidak's post‐tests

The activation of GLP‐1 receptors has been reported to inhibit thermal hyperalgesia and mechanical allodynia in neuropathic rats through spinal expression of IL‐10 and subsequent β‐endorphin.^23^, ^29^, ^31^ To identify the original role of IL‐10/β‐endorphin expression involved in maladaptive synaptic plasticity, which accounts for the GLP‐1 receptor agonist exenatide‐exerted pain anti‐hypersensitivity, the IL‐10 antibody as well as β‐endorphin antiserum and CTAP were applied. The intrathecal injection of exenatide (100 ng) produced significant inhibition of thermal hyperalgesia and mechanical allodynia in neuropathic rats, but not in sham rats. Pretreatment with the intrathecal IL‐10 antibody (2 μg), β‐endorphin antiserum (1:10 dilution), and CTAP (10 μg) completely attenuated exenatide‐induced inhibition of mechanical allodynia and thermal hyperalgesia, and the baseline pain hypersensitivity response in neuropathic rats was not altered 1 h after the second intrathecal administrations (Figure 3A, mechanical allodynia, *F*_(11,354)_ = 1453, *p* < 0.0001, two‐way repeated‐measures ANOVA followed by Sidak's post‐tests; thermal hyperalgesia, *F*_(11,59)_ = 113.3, *p* < 0.0001, one‐way ANOVA followed by Sidak's post‐tests).

Furthermore, the mEPSCs were recorded in the lamina II spinal dorsal horn neurons of both sham and SNL rats (Figure 3B). Nerve injury significantly increased the frequency and amplitude of mEPSCs, both of which were fully rescued by the bath application of exenatide (1 μM). Pretreatment with the β‐endorphin antiserum (1:300 dilution), IL‐10 antibody (4 μg/ml) and CTAP (1 μM) did not significantly alter baseline transmission of sham rats, but entirely reversed exenatide‐induced suppression of mEPSCs in neuropathic rats (Figure 3C,D, Frequency, *F*_(11,95)_ = 15.89, *p* < 0.0001; Amplitude, *F*_(11,95) =_28.17, *p* < 0.0001, one‐way ANOVA followed by Sidak's post‐tests).

The mIPSCs were also recorded in the spinal dorsal horn of lamina II neurons from sham and neuropathic rats (Figure 3E). The frequency, but not the amplitude, of mIPSCs was reduced in the SNL group. Bath application of exenatide (1 μM) did not significantly suppress the frequency or amplitude of mIPSCs in either neuropathic or sham rats (Figure 3F,G, Frequency, *F*_(3,33)_ = 19.68, *p* < 0.0001; Amplitude, *F*_(3,33)_ = 1.220, *p* = 0.3177, one‐way ANOVA followed by Sidak's post‐tests).

### 3.4 Exogenous β‐endorphin ameliorated pain hypersensitivity and spinal synaptic excitatory transmission.

**FIGURE 4:**
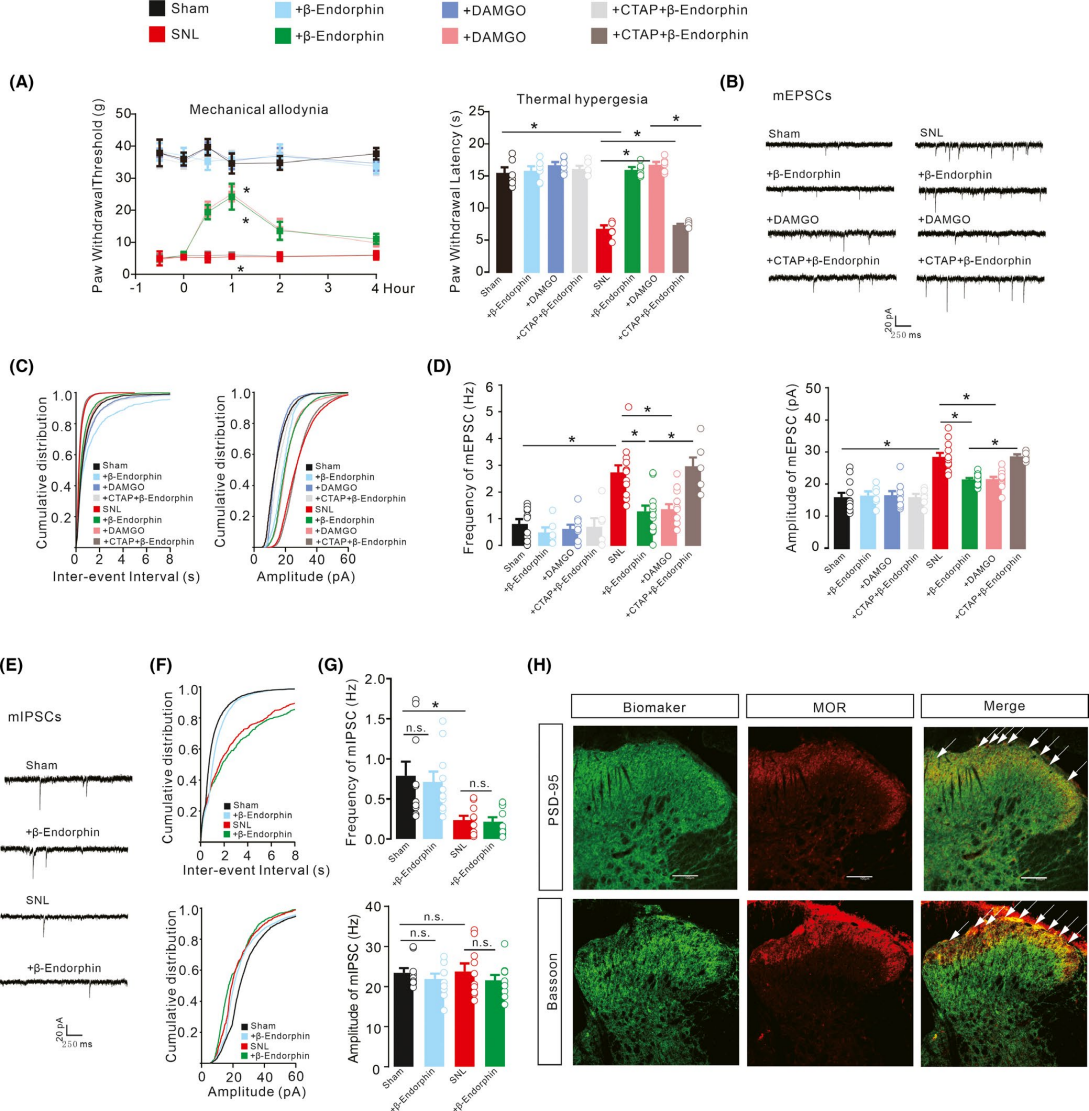
Blockade effects of the μ‐opioid receptor (MOR) antagonist CTAP on β‐endorphin and DAMGO‐induced mechanical antiallodynia and thermal antihyperalgesia in the ipsilateral hind paws (A), inhibition of spinal excitatory synaptic transmission (B‐D), and effects on inhibitory synaptic transmission (E‐G) in neuropathic rats. Neuropathic rats were induced by ligation of spinal nerves. Representative mEPSCs and mIPSCs traces, cumulative distribution and statistical analyses were obtained from the lamina II spinal dorsal horn neurons. Scale bars, 40 pA and 10 s. Representative double immunofluorescence staining of MORs with the presynaptic and postsynaptic biomarkers Bassoon and PSD‐95 in the spinal dorsal horns from three normal rats (H). Scale bar: 100 μm. The data are presented as the means ± SEM (n = 6 animals per group). *p < 0.05, by one‐way or repeated‐measures two‐way ANOVA followed by Sidak's post‐tests)

The MOR is a well‐known target for pain relief.^55^, ^56^ To further confirm the role of β‐endorphin involved in IL‐10 and exenatide‐induced inhibition of spinal synaptic plasticity in neuropathic pain, exogenous β‐endorphin and another specific MOR agonist DAMGO were employed. Sham and neuropathic rats individually received ACSF (10 μl) or CTAP (10 μg) 0.5 hours later followed by intrathecal injection of ACSF (10 μl), β‐endorphin (20 μg), or DAMGO (10 μg). The intrathecal injection of β‐endorphin and DAMGO significantly inhibited mechanical allodynia and thermal hyperalgesia in neuropathic rats but not in sham rats. Pretreatment with intrathecal CTAP completely blocked β‐endorphin‐induced inhibition thermal hyperalgesia of and mechanical allodynia in neuropathic rats (Figure 4A, mechanical allodynia, *F*_(7,240)_ = 1634, *p* < 0.0001, two‐way repeated‐measures ANOVA followed by Sidak's post‐tests; thermal hyperalgesia, *F*_(7,40)_ = 61.69, *p* < 0.0001, one‐way ANOVA followed by Sidak's post‐tests).

Recordings of mEPSCs were acquired from ipsilateral slices of lamina II neurons of sham and neuropathic rats (Figure 4B). Bath application of β‐endorphin (1 μM) and DAMGO (1 μM) suppressed the increased frequency and amplitude of mEPSCs in SNL rats. Pretreatment with CTAP (1 μM) significantly blocked β‐endorphin‐inhibited frequencies and amplitudes of mEPSCs (Figure 4C,D, Frequency, *F*_(7,72)_ = 15.13, *p* < 0.0001; Amplitude, *F*_(7,72)_ = 23.63, *p* < 0.0001, one‐way ANOVA followed by Sidak's post‐tests).

The mIPSCs were further recorded in spinal dorsal horn of lamina II neurons to reflect inhibitory synaptic transmission of MOR (Figure 4E). The bath application of β‐endorphin (1 μM) did not significantly affect the frequency and amplitude of mIPSCs in the ipsilateral spinal dorsal horn neurons of the SNL rats (Figure 4F,G, Frequency, *F*_(3,36)_ = 6.860, *p* = 0.0009; Amplitude, *F*_(3,36)_ = 0.5770, *p* = 0.6339, one‐way ANOVA followed by Sidak's post‐tests).

The inhibitory effects of β‐endorphin and DAMGO on the frequency and amplitude of mEPSCs suggest that MORs may be expressed on both presynaptic and postsynaptic neurons. To confirm this, MORs were labeled through double immunostaining. Bassoon was used for presynaptic neurons and NeuN for postsynaptic neurons in the spinal dorsal horns of normal rats. As shown in Figure 4H, MORs were colocalized with pre‐ and postsynaptic neuronal biomarkers in the spinal dorsal horns, particularly in the laminae I/II.

### 3.5 Microglia mediated IL‐10‐ and exenatide‐inhibited spinal excitatory synaptic transmission in neuropathic pain.

**FIGURE 5:**
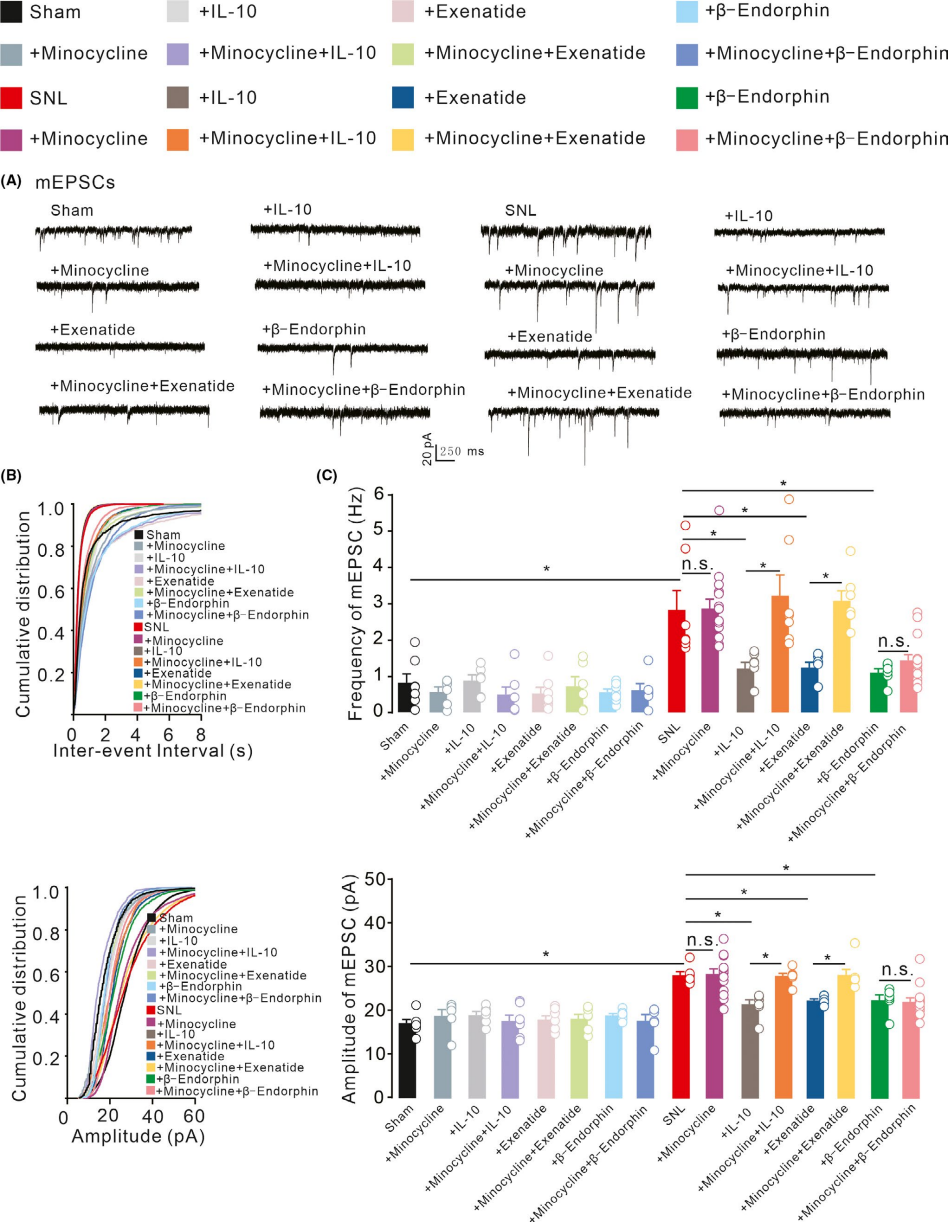
The effects of the microglial inhibitor minocycline on IL‐10‐, GLP‐1 receptor agonist exenatide‐, and β‐endorphin‐induced suppression of spinal excitatory synaptic transmission in neuropathic rats (A‐C). Neuropathic rats were induced by ligation of spinal nerves. Representative mEPSCs recording traces, cumulative distribution and statistical analyses were obtained from the lamina II spinal dorsal horn neuron. Scale bars, the data are presented as the means ± SEM. *p < 0.05, by one‐way ANOVA followed by Sidak's post‐tests

Exenatide and IL‐10 have been reported to alleviate pain hypersensitivity via the microglial expression of β‐endorphin, but not via astrocytes or neurons.^29^, ^30^, ^31^, ^57^ To examine whether microglia mediated exenatide‐ and IL‐10‐induced inhibition of spinal synaptic plasticity in neuropathic pain, the microglial inhibitor minocycline was applied. Bath application of exenatide (1 μM), IL‐10 (100 ng/ml), and β‐endorphin (1 μM) significantly reduced the increased mEPSCs frequency and amplitude of the neuropathic rats. Pretreatment with minocycline (100 μg) did not significantly suppress baseline values of mEPSCs in sham or neuropathic rats, but entirely restored the IL‐10‐ and exenatide‐inhibited frequencies and amplitudes of mEPSCs in the neuropathic rats. However, minocycline did not significantly affect β‐endorphin‐induced inhibition of synaptic plasticity in neuropathic rats (Figure 5A‐C, Frequency, *F*_(15,108)_ = 14.34, *p* < 0.0001; Amplitude, *F*_(15,108)_ = 0.5770, *p* < 0.0001, one‐way ANOVA followed by Sidak's post‐tests).

## 4 DISCUSSION

Our study confirmed that SNL induced profound and long‐lasting thermal hyperalgesia and mechanical allodynia, accompanied by the overexpression of neuronal c‐fos, upregulation of microglia and astrocytes, and loss of neurons. Whole‐cell recordings in the ipsilateral neurons of laminae II from neuropathic rats exhibited enhanced excitatory synaptic transmission reflected by the frequency and amplitude of mEPSCs and reduced descending inhibitory synaptic transmission reflected by the frequency but not the amplitude of mIPSCs. As the enhanced spinal excitatory transmission is recognized as a critical element for neuropathic pain, we inferred that microglial IL‐10/β‐endorphin signaling linked to sufficiently and efficiently pain relief mediated by presynaptic and postsynaptic MOR.

Studies have shown a correlation between microglial expression of β‐endorphin and IL‐10 or endomorphin‐2 in depressive disorders and chronic pain.^23^, ^58^ Additionally, through spinal microglial, not astrocytic expression of β‐endorphin, IL‐10 has been shown to produce pain anti‐hypersensitivity.^23^, ^30^, ^58^ Current evidence indicates that the microglial IL‐10/β‐endorphin signaling has broad biological significance in the regulation of pain hypersensitivity in chronic pain states. GLP‐1, GPR40, and α7‐ACh receptor agonists have effectively attenuated pain hypersensitivity in neuropathic pain, inflammatory pain, bone cancer pain, and diabetic pain through spinal microglial autocrine expression of IL‐10 and subsequent expression of β‐endorphin.^30^, ^32^, ^33^, ^34^, ^35^ In addition, early life‐induced constitutive suppression of neuropathic pain has been found to be mediated by the microglial IL‐10/β‐endorphin signaling.^59^, ^60^ Moreover, electroacupuncture has suppressed hypersensitive behaviors in neuropathic pain through spinal microglial expression of IL‐10.^61^ However, the underlying mechanism of IL‐10 signaling has not been thoroughly elucidated from synaptic connections. Our current study further confirmed that IL‐10/β‐endorphin expression in exenatide‐induced inhibition of spinal synaptic plasticity and pain hypersensitivity in neuropathic rats. However, activation of GLP‐1 receptors was previously reported to enhance the depolarization‐evoked release of glutamate and GABA in the mouse cortex and hippocampus. ^62^, ^63^

Upon confirming the inhibitory effect of the β‐endorphin antiserum and MOR antagonist CTAP on spinal IL‐10‐induced thermal antihyperalgesia and mechanical antiallodynia, we focused on their blockade effects on IL‐10‐induced inhibition of spinal synaptic plasticity. Bath application of IL‐10 reduced enhanced frequencies and amplitudes of mEPSCs in the spinal dorsal horn neurons of laminae II from neuropathic rats to the level of the sham rats, which were in agreement with the previous study that IL‐10 instead facilitated both presynaptic and postsynaptic transmission and homeostatic plasticity in cultured hippocampal neurons.^62^, ^63^ In contrast, IL‐10 did not significantly affect reduced frequencies of mIPSCs in the spinal dorsal horn neurons of laminae II from neuropathic rats. The results suggest that IL‐10 reduces excitatory synaptic transmission but not descending inhibitory transmission, which contributes to its pain anti‐hypersensitivity.

Pretreatment with β‐endorphin antiserum could totally rescue IL‐10‐induced inhibition of the elevated frequencies and amplitudes of mEPSCs in neuropathic rats, but not in naive rats. The results indicated that IL‐10 reduces excitatory synaptic transmission by regulating β‐endorphin expression, which is confirmed by the finding that exogenous β‐endorphin inhibited the frequencies and amplitudes of mEPSCs in neuropathic rats. In addition, MORs are indirectly associated with IL‐10‐induced inhibition of spinal synaptic plasticity, as β‐endorphin has a high affinity for MORs. The specific MOR agonist DAMGO has a similar inhibitory effect as IL‐10 or β‐endorphin, whose effects were blocked by the specific MOR antagonist CTAP in neuropathic rats. Neuronal MORs predominately mediate inhibitory effects on neuronal activity, which mainly related to decrease intracellular adenylyl cyclase activity and involved in voltage‐gated N‐type calcium currents in spinal neurotransmission.^4^, ^64^, ^65^

Our study extensively supported that IL‐10 reduced excitatory synaptic transmission through presynaptic and postsynaptic MOR mechanisms. Numerous studies have also identified presynaptic and postsynaptic MORs involved in synaptic transmission.^66^ The application of DAMGO led to the presynaptic inhibition of primary afferent neurons and decreased the frequencies of mEPSCs via the activation of presynaptic MORs.^4^, ^66^, ^67^ MORs also serve as the crucial role in postsynaptic transmission, as bath application of DAMGO induces an directly outward current mediated by activation of K^+^ channels and reduces the amplitude of EPSCs of GABAergic interneurons which might receive monosynaptic inputs from primary nociceptive C fibers, and the downregulation of MORs could weaken the inhibition of postsynaptic neurons excitability, which could be explained by hypersensitivity in pain states.^66^, ^67^ Indeed, our data showed that IL‐10 and β‐endorphin inhibited excitatory transmission mediated by presynaptic and postsynaptic MORs in spinal cord, and using double immunohistochemical staining, MORs were significantly colocalized with bassoon and NeuN in laminae I/II. It is widely accepted that bassoon serves as the presynaptic marker; NeuN were only applied for biomarker for neurons. In our study, whole‐cell recording the neurons of laminae II were attached for electrophysiological analysis and served as the postsynaptic. Therefore, NeuN or PSD‐95 chosen for postsynaptic marker in our study.

Spinal excitatory transmission served as crucial elements in rats subjected to peripheral spinal nerve ligations; meanwhile, SNL‐induced inflammatory derived responses and enhanced excitatory transmission. The excessive and long‐lasting excitatory transmission from DRG exhibited, shown a significantly neuronal loss in spinal dorsal horn of laminae II from neuropathic rats and remarkable neuronal organization and synaptic arrangements.^68^ Disinhibition of inhibitory transmission closely related from descending inhibitory system were also arisen and also implicated the trans‐synaptic effects induced by enhanced excitatory transmission. In behavioral tests, no matter the drugs applied, mechanical thresholds of neuropathic rats could not totally reverse, it might be inferred that those impairments induced neuronal organization and synaptic arrangements might be implicated in the behavioral observations.

In addition, our previous study manifested that dose‐dependent injection of IL‐10 and exenatide could reduce pain states in rats. It might be inferred that agonists, we applied in our study, have been extensively studied by using dosage‐dependent application in behavioral and primary culture tests.^30^, ^35^ Furthermore, the antagonists were also critically identified by the minimum, and sufficient dosages applied in general.^32^, ^33^ Thus, the results are sufficient to support our results. Some studies have suggested gender difference in neuropathy of microglia, where nerve damage‐induced neuroinflammatory cytokines expression and pain like behavers were showed to be dominant in male animals. In contrast, other findings have revealed that no sex dimorphism is found in microglial mediation of anti‐hypersensitivity effects on neuropathic pain conditions such as gabapentin, IL‐10, electroacupuncture, α7nAChR agonists, and/or genetic deletion of the microglia selective molecules involving CX3CR1, P2Y12, and TMEN16F.^23^, ^34^, ^61^, ^69^, ^70^, ^71^, ^72^

It is clear that the strengthened neuronal connections between primary afferents and dorsal horn neurons have led to a state of hyperalgesia through sensory circuits in the spinal dorsal horn, accompanied by an imbalance between glutamatergic and GABAergic/glycinergic transmissions.^51^ However, activation of microglia and interactions between microglia and neurons might shape the function and synaptic circuits in neuropathic pain. At the early stage of neuropathic pain, microglia‐derived inflammatory cytokines, including IL‐6, IL‐1β, and TNF‐α, participate in synaptic plasticity by modulating the functions of synaptic receptors, channels, and enzymes such as voltage‐gated Ca^2+^ channels, the Src family of kinases, NMDA receptors, and metabotropic glutamate receptors, which eventually potentiated neuronal transmission of pain messages to higher neurons.^36^, ^73^, ^74^, ^75^, ^76^, ^77^, ^78^ Meanwhile, the CXCL12/CXCR4 signaling pathway also contributed to neuropathic pain through regulation of central sensitization.^79^ Silencing of microglia‐specific Ca^2+^‐activated K^+^ auxiliary β3 subunit significantly increases neurotransmission and attenuates antinociceptive tolerance.^80^ Additionally, the regulation of microglia‐specifically expressed P2Y12 reduces inflammation, pain sensation, and related glutamatergic transmission in spinal dorsal horn.^81^ In contrast, our data provided evidence that activation of microglial IL‐10 and GLP‐1 receptors inhibited the frequencies and amplitudes of mEPSCs via the expression of β‐endorphin, which was specifically and completely abolished by the microglial metabolic inhibitor minocycline, although minocycline did not affect β‐endorphin‐inhibited neurotransmission. In addition, *aconitum*‐derived bulleyaconitine A was recently reported to stimulate microglia to release dynorphin A, which specifically activates presynaptic κ‐opioid receptors in afferent neurons of substantia gelatinosa (SG) and inhibits spinal synaptic plasticity.^82^ These results could provide evidence for interactions between microglia and neurons through endogenous peptides, which might primarily inhibit spinal synaptic plasticity and pain transmission and transduction.

## 5 CONCLUSIONS

**FIGURE 6:**
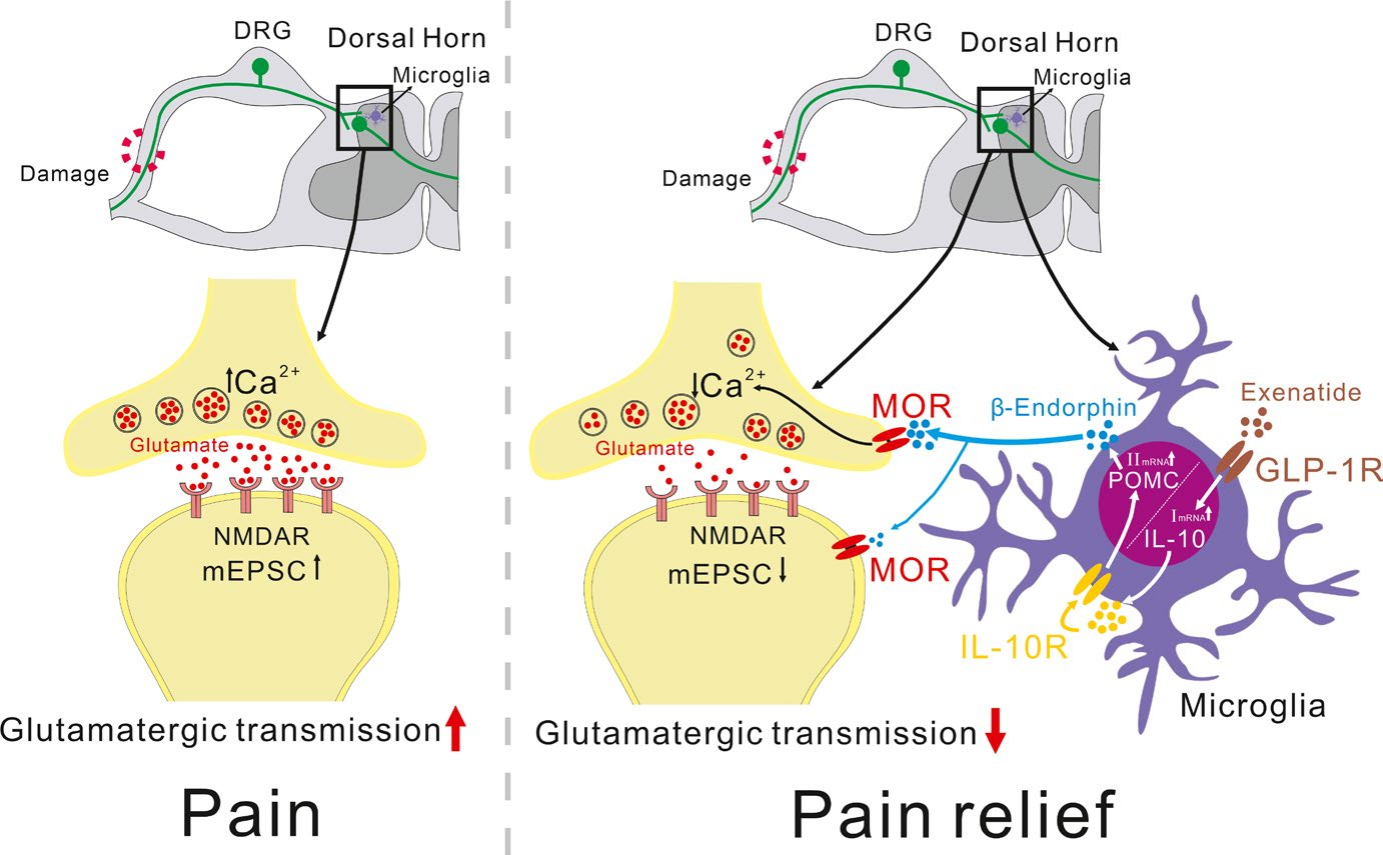
Schematic diagram showing the role of microglial expression of β‐endorphin in IL‐10‐ and specific GLP‐1 receptor agonist exenatide‐induced inhibition of spinal excitatory synaptic transmission and pain hypersensitivity in neuropathic pain. Following activation of GLP‐1 receptors, IL‐10 is released and then activates IL‐10 receptors via a microglial autocrine mechanism. Afterward, the β‐endorphin is released to microglial neuronal synapses and activates neuronal presynaptic and postsynaptic μ‐opioid receptors (MORs) to inhibit the enhanced glutamatergic transmission, leading to pain anti‐hypersensitivity

Our results have illustrated that treatment with IL‐10 and exenatide produces pain anti‐hypersensitivity in neuropathic rats and inhibits pre‐ and postsynaptic glutamatergic transmission in the spinal dorsal horn, which was blocked by minocycline, β‐endorphin antiserum, and CTAP. Figure 6 schematically presents the proposed role of microglial expression of β‐endorphin through autocrine IL‐10‐ and exenatide‐induced inhibition of spinal synaptic plasticity and pain anti‐hypersensitivity in neuropathic pain. Our results provide evidence that activation of microglial IL‐10/β‐endorphin signaling contributes to pain management and amelioration of maladaptive circuits in neuropathic pain.

## CONFLICT OF INTEREST

The authors declare that there are no competing financial interests in this work.

## AUTHORS’ CONTRIBUTIONS

YXW, JHC, and LM conceived and designed the experiments. LM, SYP, JBW, MJZ, and KAA performed the experiments. LM and YXW analyzed the data and involved in preparation of the article. All authors discussed and revised the manuscript and approved the final manuscript.

## ETHICS APPROVAL STATEMENT

All experiments were performed in accordance with the Animal Care and Welfare Committee of Shanghai Jiao Tong University and followed the animal care guidelines of the National Institutes of Health.

## Data Availability

All data, models, or figures generated or used during the study are available from the corresponding authors.
